# Integrating participant feedback and concerns to improve community and individual level chemical exposure assessment reports

**DOI:** 10.1186/s12889-023-16661-0

**Published:** 2023-09-06

**Authors:** Samantha M. Samon, Michael Barton, Kim Anderson, Abiodun Oluyomi, Melissa Bondy, Georgina Armstrong, Diana Rohlman

**Affiliations:** 1 Department of Environmental & Molecular Toxicology, Oregon State University, Corvallis, OR USA; 2 Pacific Northwest Center for Translational Environmental Health Research, Oregon State University, Corvallis, OR USA; 3 Section of Epidemiology and Population Sciences, Department of Medicine, Baylor College of Medicine, Houston, TX USA; 4 Gulf Coast Center for Precision Environmental Health, Baylor College of Medicine, Houston, TX USA; 5 Department of Epidemiology and Population Health, Stanford School of Medicine, Stanford University, Stanford, CA USA; 6 College of Health, Weniger Hall 223, 103 SW Memorial Place, Corvallis, OR 97331 USA

**Keywords:** Report back of research results, Environmental health literacy, Silicone wristbands, Community-engaged research

## Abstract

**Background:**

As exposure assessment has shifted towards community-engaged research there has been an increasing trend towards reporting results to participants. Reports aim to increase environmental health literacy, but this can be challenging due to the many unknowns regarding chemical exposure and human health effects. This includes when reports encompass a wide-range of chemicals, limited reference or health standards exist for those chemicals, and/or incompatibility of data generated from exposure assessment tools with published reference values (e.g., comparing a wristband concentration to an oral reference dose).

**Methods:**

Houston Hurricane Harvey Health (Houston-3H) participants wore silicone wristbands that were analyzed for 1,530 organic compounds at two time-points surrounding Hurricane Harvey. Three focus groups were conducted in separate neighborhoods in the Houston metropolitan area to evaluate response to prototype community and individual level report-backs. Participants (*n* = 31) evaluated prototype drafts using Likert scales and discussion prompts. Focus groups were audio-recorded, and transcripts were analyzed using a qualitative data analysis program for common themes, and quantitative data (ranking, Likert scales) were statistically analyzed.

**Results:**

Four main themes emerged from analysis of the transcripts: (1) views on the report layout; (2) expression of concern over how chemicals might impact their individual or community health; (3) participants emotional response towards the researchers; and (4) participants ability to comprehend and evaluate environmental health information. Evaluation of the report and key concerns differed across the three focus groups. However, there was agreement amongst the focus groups about the desire to obtain personal exposure results despite the uncertainty of what the participant results meant.

**Conclusions:**

The report-back of research results (RBRR) for community and individual level exposure assessment data should keep the following key principles in mind: materials should be accessible (language level, data visualization options, graph literacy), identify known information vs unknown (e.g., provide context for what exposure assessment data means, acknowledge lack of current health standards or guidelines), recognize and respect community knowledge and history, and set participant expectations for what they can expect from the report.

**Supplementary Information:**

The online version contains supplementary material available at 10.1186/s12889-023-16661-0.

## Background

Exposure to environmental chemical hazards is ubiquitous in the modern era. Exposure assessments are often needed to determine the extent of chemical exposure present in a population [1]. Exposure assessments typically involve measuring levels of chemicals in biological samples (e.g., blood or serum), environmental samples (e.g., soil, water, air, food), or noninvasive personal exposure assessment tools (e.g., silicone wristbands, hand wipes, household dust) [1–4]. A communication gap often exists between the researchers and the participants or affected communities, particularly when communicating the results of the research and their relationship to health [5]. Report back of research results (RBRR) is a potential avenue to reduce the gap between exposure assessment researchers and the general population [6]. RBRR can refer to the return of research results to an individual participant and/or the larger community [7, 8]. Results reported back may include contaminant levels measured in personal exposure assessments or environmental samples collected from that individuals’ home, while community level reports focus on the de-identified, aggregate results from the study [7]. The practice of RBRR is rapidly becoming considered an ethical obligation [9], despite early concerns of: i) reporting back information without clear health guidelines [6, 11, 12]; ii) results that may cause participants to worry or change their behavior in detrimental ways; [12–14], and; iii) potential legal considerations, (e.g., when needing to disclose well water contaminants during the sale of a house) [12]. Yet, research has shown that participants are not overly alarmed by their results and generally wanted their results regardless of the potential negative emotions and/or legal requirements that come with it [12, 16, 19].

Furthermore, RBRR can lead to increased environmental health literacy (EHL) [11, 20, 21], another approach for reducing the communication gap. At its most fundamental level EHL involves “an understanding of the connection between environmental exposures and human health” [22]. Reporting environmental assessment data back to research study participants has shown to be a successful way to increase participants’ level of understanding regarding their risks and can empower participants to exert control over environmental exposures that may lead to adverse health outcomes [5, 16, 20, 21]. This can in turn lead to individual, community, or policy level changes to reduce chemical exposure [5, 11, 12, 16, 17, 20–23].

There are current evidence-based practices for conducting exposure assessment data report-back, such as the Clear Communication Index [24], the Handbook for Reporting Results to Participants [7], and many theoretical models for EHL and communication [22]. Further development for messaging and evaluation are still needed to increase accessibility and usability of RBRR. Thus far case studies and evaluations of exposure assessment report-backs have focused on evaluating changes in EHL via pre/post surveys, interviews, and focus groups [11, 16, 21, 25], but there are minimal examples wherein participants’ preference for data visualization is assessed, particularly in instances where the environmental contaminants lack regulatory or health standards, or a clear relationship to health outcomes.

As part of the Houston Hurricane Harvey Health (Houston-3H study), participants wore silicone wristbands to capture personal chemical exposure, and had the option to receive their individual results. Silicone wristbands are easy to use and minimally invasive [26, 27]. Because of their placement on the wrist, silicone wristbands capture dermal exposure, exposure via inhalation, and compounds that are excreted through the skin [27, 28]. While easy-to-use, reporting data back from the wristband can be challenging beyond the expected challenges of communicating scientific data. In the Houston-3H study, over 1,530 chemicals were assessed, and most of the chemicals assessed lack regulatory and health standards or guidelines. For chemicals that would be considered clinically actionable, the wristband data, reported in amount of chemical per wristband, is not currently comparable to reference values, although there are efforts underway to bridge this [27, 28]. While this limitation is common to many exposure assessment tools, the number of chemicals with no regulatory information further complicates how to relate exposure to health in study report backs.

Reporting-back data from exposure assessment studies is a meaningful opportunity to increase EHL and provide report recipients with valuable information relevant to their personal and environmental health [11, 21]. However, this opportunity only exists when the materials are developed in a manner that is accessible and appropriate for the audience and account for the limitations that currently exist in exposure assessment. In this study researchers piloted RBRR materials from an exposure assessment study conducted after Hurricane Harvey using silicone wristbands. The purpose of this study was to get feedback from people unfamiliar with exposure assessment on how best to report data in the absence of clinical significance or regulatory guidelines and create a set of recommendations to aid in future report-back generation.

## Participants & methods

Focus groups to evaluate presentation of personal chemical exposure data were conducted as part of the Houston-3H project. Full details of the Houston-3H project are described by Oluyomi et al. [31]. Briefly, participants were recruited from neighborhoods in Harris County, TX (e.g., Addicks, Baytown, Bellaire-Meyerland, East Houston) that were heavily impacted by Hurricane Harvey flooding. Eligibility criteria included being age five or older, and fluent in English and Spanish. Overall, the project aimed to evaluate exposure to chemical and microbial contaminants following Hurricane Harvey and the potential impacts on health [31, 32]. To evaluate individual-level chemical exposure, participants wore silicone wristbands for a seven-day time period during the first round of sampling (September 23—October 12, 2017), which occurred within one month of flooding from Hurricane Harvey, and during a second round of sampling approximately one year later (September 18–27, 2018). In total, 312 participants wore and returned a silicone wristband at one or both time points. Silicone wristbands were analyzed using gas chromatography-mass spectroscopy with a screening method for 1,530 organic chemicals to capture a broad range of potential environmental contaminants [31, 32]. The Houston-3H study was approved by the institutional review boards at Oregon State University, Baylor College of Medicine (BCM), and the University of Texas Health Science Center. All participants had the option during consent to receive the results from their silicone wristband, and 100% requested their results.

### Example report generation

**Fig. 1:**
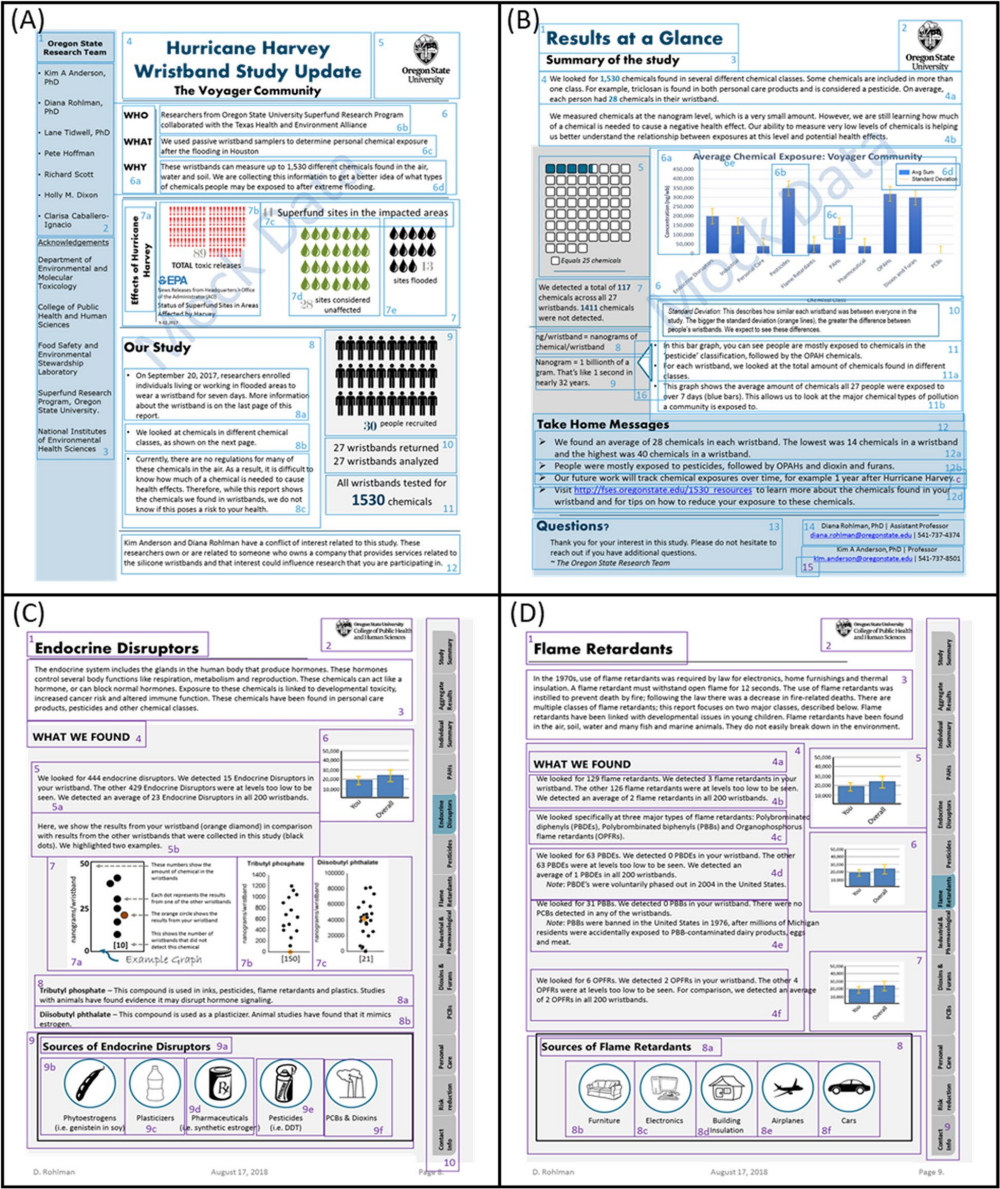
Example study reports distributed at the focus group for review. Example reports included a two-page community report (**A** and **B**) and two pages taken from the example individual report (**C** and **D**). Individual report pages reviewed included a page dedicated to endocrine disruptors (**C**) and flame retardants (**D**). After reports were presented at the focus groups they were divided into subsections to quantitatively review participant likes and dislikes

Example community and individual level reports were generated using mock data and fictional communities. Real data were not used as analysis was on-going and mock data allowed for an unbiased approach to the data visualization. The example reports were screened using the CDC Clear Communication Index [24], the Flesch Kincaid Grade Level Score [33] (desired level of 8^th^ grade; scores ranged from 6.6–12.4) and the Flesch Reading Ease formula (desired score of > 60%; scores ranged from 45–53) [34] using the built-in tools in Microsoft Word. The Clear Communication Index uses evidence-based communication strategies to score communication products, while the Flesh Kinkaid and Flesch Reading Ease metrics are commonly used measures of readability in health care [35]. The report consisted of the following 12 pages, although the focus groups reviewed only the community report and two individual chemical pages (Fig. 1):

- Community report (2 pages). Provided a brief study overview and interpretation of the aggregated, de-identified data.
- Individual Data (9 pages). Given the number of chemicals assessed, the report binned chemicals into nine chemical categories based either on common use (e.g., flame retardants), chemical structure, or biological effect (e.g., endocrine disruptor). Each of the nine chemical categories (polycyclic aromatic hydrocarbons, endocrine disruptors, pesticides, flame retardants, industrial, pharmacological, dioxins/furans, and personal care products) was described in a single page. A simple description of the chemical category was followed by the individual’s results, placed in the context of the study population.
- Full Individual results (2–3 pages). A table containing all the chemical detections specific to an individual, by chemical categorization, completed the report.

### Recruitment and participants

All Houston-3H participants were contacted by email to elicit interest in the focus groups. Those who expressed interest were later contacted by phone to remind them of the upcoming focus group, and reiterate the goals of the focus group. Thus, focus groups were conducted at several study locations representative of the original Houston-3H study recruitment sites. In total three separate focus groups were held: one at Baylor College of Medicine to represent the Bellaire-Meyerland Neighborhood, and one each at community centers located within the Addick’s and Baytown neighborhoods. Due to low interest from participants living within the East Houston neighborhood, a separate focus group was not held within this neighborhood. Participants living within the East Houston neighborhood were instead invited to the Addicks focus group. Each focus group session included a complementary meal, and participants received a $20 gift card to a local grocery store.

### Focus group procedure

Prior to the focus groups, members consented to participation which included audio recordings and deidentified transcriptions. Focus groups consisted of semi-structured 90-min in-person sessions moderated by a group leader and a note taker. Each participant was given a packet upon entry that contained the prototype community and individual level reports and a survey containing questions related to demographic, preferences/rankings for different data visualization styles, and multiple Likert scale questions concerning report usefulness. Prototypes consisted of a two-page community report and two pages taken from the example individual report, including an overview page about endocrine disruptors and flame retardants (Fig. 1).

Evaluation of example community and individual level report-backs was comprised of multiple components. Participants identified elements of the reports they liked or disliked by circling them in black or red, respectively, allowing for quantitative evaluation, and then discussed their preferences and provided a score (1–10) for the community report and both example pages of the individual report. Additionally, ranked choice questions were used to assess preferences for graph types and data visualization.

### Analysis

#### Qualitative analysis

Analysis utilized a mixed methods approach, which included both quantitative and qualitative analysis. All focus groups were audio recorded and transcribed for coding. After cross-checking the transcripts against the records, and verifying the accuracy of the transcripts, the transcripts were analyzed, and a thematic coding framework was developed and applied. Analysis followed both a thematic and pragmatic approach [36], whereby review of the transcripts informed the inductive generation of themes, and initial broad research questions informed the deductive generation of themes. Initial primary codes were developed and connected to other related codes to form secondary codes that were developed into four themes. Data collection and analysis continued until saturation occurred (i.e., until no new significant themes emerged). Data were analyzed in NVivo (V.12.4.3, QSR).

#### Quantitative analysis

The prototype report-backs including the community report and the example individual report pages were divided into sections and subsections to better quantitate what participants circled in black and red (Fig. 1). Key likes and dislikes were identified based on the percentage of participants who circled that section or subsection.

## Results

### Description of the study population

Focus group sizes ranged from nine to thirteen participants. Relative to the total Houston-3H population that wore and returned wristbands, there was a higher percentage of senior participants in the focus groups, with no participants under the age of 34 (Table 1). The education level of focus group participants was slightly higher from the Houston-3H population. Focus groups had a higher proportion of individuals with an advanced degree, and a lower portion of individuals with a high school education or less compared to the Houston-3H population. Lastly, focus groups had fewer participants who identified as Latino (Table 1). The race/ethnicity and education levels varied largely by focus group location. The majority of participants in focus groups held at Baylor College of Medicine and the Addick’s neighborhood identified as white and had a bachelor’s degree or higher. In contrast, the focus group held in Baytown consisted mostly of participants who identified as Black/African American and had less than a bachelor’s degree (Table 1).

**Table 1:** Participant demographics for the aggregate focus group participants, and focus groups held at Baylor College of Medicine, the Addick’s neighborhood, and the Baytown neighborhood

Demographics	Baylor	Addick’s	Baytown	Aggregate Focus Groups		Houston 3-H Population^a^	
	N	N	N	N	%	N	%
Gender							
Female	9	9	6	24	77.4	208	66.7
Male	0	4	3	7	22.6	104	33.3
Age Group							
Youth (<18)	0	0	0	0	0	31	9.9
Adult^b^	6	4	4	14	45.2	190	60.9
Senior (> 64)	3	9	5	17	54.8	87	27.9
Prefer not to Answer	0	0	0	0	0	4	1.3
Race/Ethnicity							
Asian	2	0	0	2	6.45	22	7.1
African American	0	1	8	9	29.0	106	34.0
Latino	0	1	1	2	6.45	63	20.2
White	7	11	0	18	58.1	115	36.9
Multiracial	0	0	0	0	0	6	1.9
Education Level							
High School	0	0	3	3	9.7	93	29.8
Some College	0	0	4	4	12.9	38	12.2
Associate Degree ^c^	0	2	1	3	9.7	83	26.6
Bachelor’s Degree^c^	2	4	1	7	22.6		
Master’s Degree^c^	2	5	0	7	22.6	90	28.8
Doctoral Degree^c^	5	2	0	7	22.6		
Prefer not to Answer	0	0	0	0	0	8	2.6
Overall	9	13	9	31	-	312	-

^a^This represents the Houston-3H population that wore and returned a wristband at one or both timepoints and received community and individual level reports.; ^b^Of note, the youngest participant in the focus groups was 34 years of age at the time the focus group was held. All ages were adjusted to represent approximate age at the time focus groups were held; ^c^Houston-3H questionnaires only included one “undergraduate” option and one “advanced degree” designation for education level and the proportion of Houston 3-H participants who had associates versus bachelor’s degrees and masters versus doctoral degrees is not known

### Qualitative analysis

To gain a deeper understanding of what information participants would like to know and how they would prefer data to be presented, focus group transcripts were inductively evaluated and three main themes emerged: (1) Feedback on the report layout including the appropriate use of images, colors, and language level; (2) Concern over exposure and how exposure to chemicals might impact individual or community health; and (3) participants trust and distrust of the research and/or researchers. A fourth theme was deductively generated to assess participants ability to comprehend and evaluate environmental health information, a component of EHL.

### Report layout

The primary goal of the focus groups was to receive feedback on the report layout, language used, and data visualization options. Such feedback would improve the final report, but importantly would ensure that RBRR was conducted in a way that is most accessible and useful to the study participants. Generally, participants thought the layout of the community report aided their understanding, read well, and had a “good balance between the graphics and the explanation.” The use of graphics were described as “easy to see and digest,” and made the information more accessible for non-experts. In addition to the graphics, the report used a section for ‘take-home messages’ which interpreted the results of the study by the researchers as four overarching points. More than 50% of the participants also indicated the take-home message was clear and easy to understand. While the layout of the report was predominantly in narrative form, one or two participants in each focus group requested the report use bulleted text rather than paragraphs.

Following the two-page community report, participants reviewed two individual report pages, which represented how data for each chemical category could be visualized. Each page used a different layout (Fig. 1). The layout of the “Endocrine disruptors” page was preferred over the “Flame Retardant” page, mainly due to less text, and the use of dot plots over bar graphs. Specifically: “they [the dot plots] are very personalized, while conveying all the information without the need of confusion for error bars.” One participant described their preference as the following:

*I like the way the [endocrine disruptor] page is set up, I like the definition on top it explains to me what I am going to read next. I like the mixture between some of the graphs, some of the easy pictures to understand on the bottom, it’s not a lot of text. So, your eye goes there, and you kind of understand it. Versus [the flame-retardant page] that has a lot of text. So, [the endocrine disruptor page] had a really good mixture of the graphical and written information.*

Feedback on the readability of the community and individual reports was varied. Participants in all three of the focus groups (39%) raised the idea of adding a glossary in the report since they were unfamiliar with some of the terminology used such as a “Superfund Site”. Participants within the focus groups at Addick’s and Baylor questioned the audience of the report, for example asking, “Does this information go out to the general public? Because the general public is not educated on that level.” While none of the participants expressed difficulty in understanding the community level report, they did raise readability concerns regarding the individual report, largely due to the more complex graphs and chemical-specific information.

Inherent to the concerns around understanding and readability were larger questions about the choice to identify two chemicals for each category. The participants grappled with the number of chemical data points (1,530 chemicals were assessed), and their desire for all of the information possible without being overwhelmed with the data.

As one participant said,

*I know this probably isn't feasible if you've got lots and lots of compounds, but I think I'd like to know, well, at least why the two that were chosen are chosen. Were they the ones that had the highest results or something? Or if possible, I'd kind of like to see the results for everything.*

#### Concern

When reviewing the prototype reports all participants expressed concern regarding chemical exposure, and 35% of all participants voiced concerns regarding how their chemical exposure could impact their individual health or the health of their community. Participants struggled to understand what reported concentrations in the wristband meant for their own health, and despite multiple statements that the concentrations found in the wristband could not be compared to a “safe level” seven participants from focus groups held at Baylor and Addick’s asked for a regulatory value to compare their results to, suggesting: “If you could add a line that says “this is a cautionary level” or “a level of concern” that we could convey to everybody.” While participants were generally interested in the context that was provided and understood the rationale behind collecting this type of data, a majority of participants wanted a clearer answer for what their chemical exposure results meant in terms of their health.

*But the meaning of the data is what we're all asking for. And you can't tell us that. So, you're trying to communicate what you found without actually being able to give us a bottom line. Which is, I think a little sad, but, we basically are contributing to the development of the database that will let us know in the future. So that's good.*

Despite these concerns regarding a lack of clinical significance, all participants expressed interest in receiving their results: “You don't want to know but you want to know. I want to know [my results]”. While concern over a lack of clinical significance was shared between focus groups, the Baytown focus group was unique for voicing concern regarding their community specifically: “I don’t care about nobody else’s community… I’m worried about my community.” In contrast, participants from the focus group held at Baylor and the Addicks neighborhood had a greater focus on their individual exposure, or an interest in how home flooding impacted chemical exposure.

#### Trust and distrust in the research

Participants from all focus groups expressed appreciation that the research was being conducted, and that they were being asked for feedback on the report. While the Addick’s and Baylor focus groups did not express distrust of the research or researchers, this was a theme identified in the Baytown focus group. Distrust initially stemmed from researchers being outsiders to the community, the conflict-of-interest statement within the report, and concerns regarding the integrity of the research being done. For the latter, a large screening method was utilized for analysis and over half of the Baytown participants expressed concern that the methodology was deliberately selected to downplay chemical exposure within the neighborhood. As one participant expressed: “I believe 1,530 chemicals is too much to be testing for”, citing concern that few chemicals would be detected in such a large screen, therefore suggesting that chemical exposure was minimal in their neighborhood. Another participant elaborated further, expressing concern over the validity of the study, since the results may interfere with their lived experiences. A majority of the Baytown participants believed that “there’s a lot of chemicals, like a lot of chemicals in this area”. A few participants expressed that they worked at chemical industries within the area, so they knew they were surrounded by “dangerous chemicals,”. and therefore would be critical of the chemical exposure results if they thought the numbers were too low.

#### Environmental health literacy (EHL)

Broadly, participants understood the basic principles of EHL and recognized that chemicals can impact human health. Overall presentation of the community and individual level reports appeared to increase EHL particularly in regards to how participants would apply the information in the report to their own lived experiences. For example, 16% of participants expressed wanting maps of where toxic waste/and or industrial sites that had chemical releases were in the area: “there's not a map of any kind, and it's great to know that there are 89 total toxic releases, but it'd be kind of nice to know where they were.” Participants (16%) also expressed a desire to send a copy of their report to their congressional representative or town council members to inform policy changes.

However, participants listed specific barriers that would impede EHL. Specifically, participants described difficulty understanding the key takeaways of the report, or interpreting the results, even within communities that had a high percentage of individuals with a bachelor’s degree or higher. For example, as one participant said: “I don't know really what it's [the report] telling me, anything of value that it’s telling me” and “my question keeps being, why is it significant and what does it mean”. Over 50% of the Baytown focus group participants, and three participants in other focus groups indicated that the individual reports were not helpful: “It didn’t do nothing for me, I mean it’s like I guess maybe I don’t understand it.”

An additional challenge faced by participants was inherent to reporting back results with limited or missing regulatory or health guidelines. To address this limitation, some chemicals in the report included toxicity information gleaned from animal studies. Within the Addick’s focus group several participants expressed agreement when one member stated that they didn’t understand why animal toxicity data would be included, or how that related to them.

*The other issues are statements like the “studies with animals have found evidence that it may disrupt hormone signaling”, but in the context of what that actually means from a health standpoint, it doesn't tell me. It tells me a fact, but it doesn't tell me what the implications are. And the same thing with the “animal studies have found that it mimics estrogen”. What is the significance of that statement?*

The major concern for most participants in terms of readability dealt with graph literacy. Twenty-nine percent of all focus group participants described graphs as being “hard to register,” and that they only understood it after discussion. However, visualizations that showed a participant’s data in the context of the study population were preferred over simple bar charts.

### Quantitative analysis – improving the report

#### Participant preferences

To initiate discussion regarding the reports, participants were asked to circle items that they liked with black pens, and items they disliked or found confusing with red pens. A summary of items circled can be found in Table S1 (community report) and Table S2 (individual report pages).

For the community report, participants liked elements that spelled out the “who, what, why” of the study, sections linking to more information about the identified chemicals, and resources for reducing exposure. Over a quarter of participants liked statements acknowledging scientific limitations that prevented connections between exposure to health, and the take-home messages. When evaluating elements they disliked, while participants disliked certain words or terms, the most substantive feedback related to the types of information participants would have preferred. Where the community report emphasized environmental impacts (e.g., number of Superfund sites flooded), participants did not like that framing.

For the individual pages, graphical representations depicting sources of chemicals was well received (> 50% of participants), along with simple text descriptions of each chemical category, or highlighted chemicals (> 25% of participants). Concerns regarding graph literacy continued, as well as the difficulties in applying the individual chemical data to exposure and health, as previously discussed.

#### Report ranks and scores

Given concerns over graph literacy and methods of data visualization, participants were asked for their feedback regarding graph types (bar graphs vs stacked graph) and use of error bars. Participants were also asked their preferences regarding data analysis, as data could be shown across the entire population, within and across neighborhoods, or based on flooding status (Figure S1). There was agreement that bar graphs, using a grouped format, were preferred (71% of participants). When looking at options for data visualization, visualizing the average chemical exposure for each chemical category grouped by community (Figure S1A, option 1), was ranked the highest. However, Addick’s preferred visualizing the data by flooding status (Figure S1A, option 4), which removed an emphasis on community differences, whereas Baytown was interested in seeing chemical exposures alone (Figure S1A, option 1).

Lastly, participants were asked whether they preferred bar graphs with or without error bars. There were an equal number of participants who preferred and did not prefer error bars. When error bar preference was evaluated in association with education level a trend emerged that outside of individuals with less than a bachelor’s degree, as education level increased preference for bar graphs with error bars decreased (Figure S2).

Each participant was asked to verbally score on a scale from one to ten, with one being perfect, the community report and each of the individual report pages (Figure S3). On average, the community report was highly rated (3.25), followed by the Endocrine Disruptor page from the individual report page (3.9) and the flame-retardant page from the individual report (4.2). Distinct differences existed between the focus group locations. The focus group held in Baytown rated the community report higher than the other two focus groups (*p* < 0.05), yet rated the Endocrine Disruptor page of the individual report lower than the other two focus groups (*p* < 0.05), and rated the Flame-Retardant page of the individual report the lowest (Figure S3), citing an inability to understand the information.

Finally, participants used a Likert scale (1 = low, 10 = high) to provide feedback on the overall report, in terms of perceived usefulness of the report, as well as ability of the information in the report to help them reduce their exposure. For perceived usefulness in terms of understanding chemical exposure, the prototype pages scored a 9 (SD = 1.10), and for informing exposure reduction actions, participants scored the report as a 9 (SD = 2.02).

### Revising and disseminating the report

**Fig. 2:**
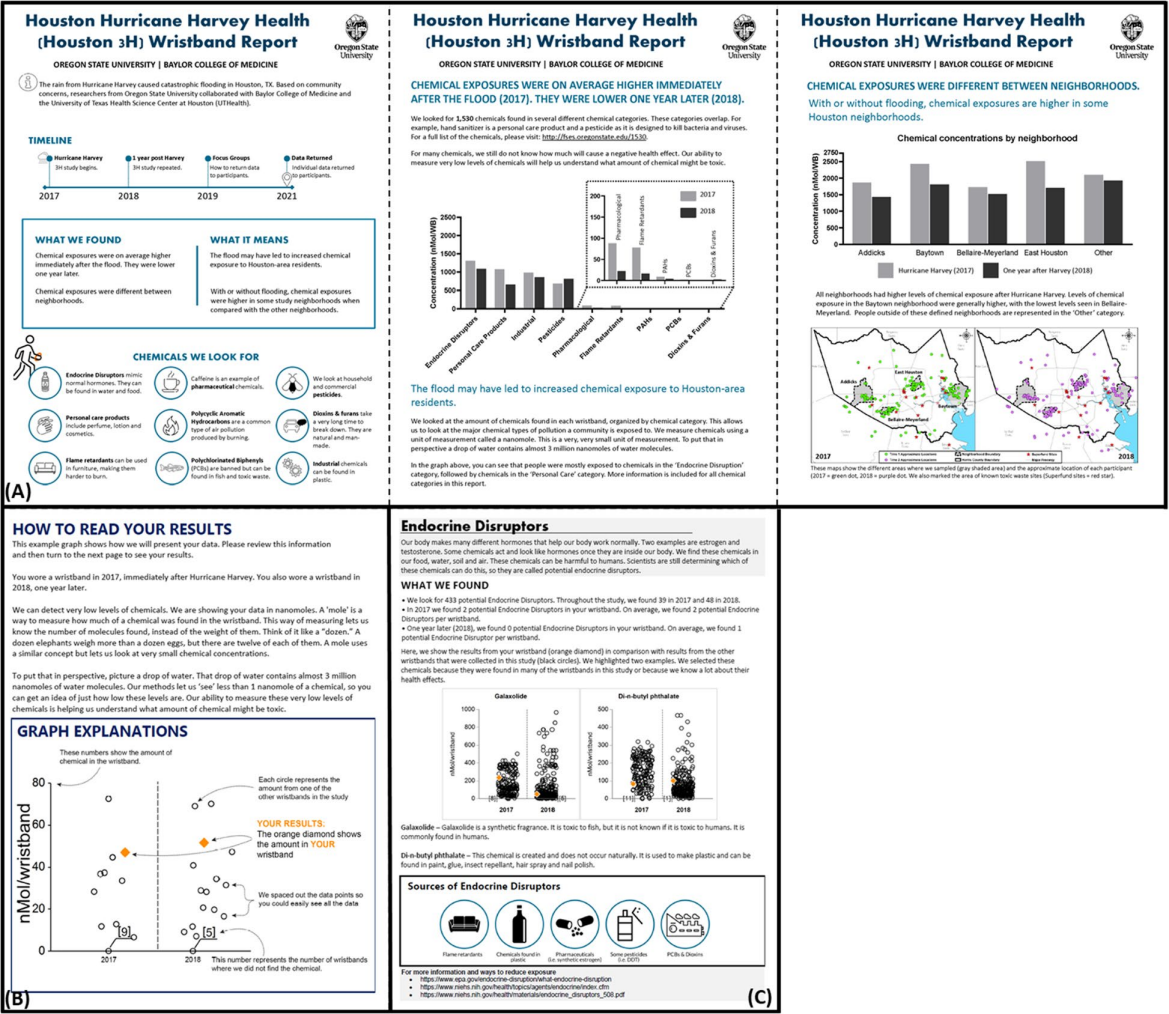
Final community report and example individual pages. The final report included a three-page community report (**A**), a page to enhance graph literacy (**B**) and a standardized individual chemical category page (the endocrine disruptor page is shown here (**C**). Reports were returned to study participants by email or mail

Following the focus groups, the chemical report (community + individual) was substantially revised and disseminated to the participants that requested their results via their preferred form of communication (email or mail). Broadly, the primary focus on the environmental impacts (e.g., number of flooded Superfund sites) was removed, and replaced with a study timeline (Fig. 2A). The take-home messages were prioritized, and simple descriptions, with graphics, of each chemical class were presented. The results were visualized by time (levels after the flood in 2017 versus levels one year later), and by community. In this way the report was able to address participant concerns regarding the impact of the flood on chemical exposures, and differences between communities. A map showing the different general sampling locations and communities was included.

To enhance graph literacy, a full page describing how to read individual results was added (Fig. 2B), where previously it had been a small figure (Fig. 1). The description included analogies to define unfamiliar units of measurement. The graph explanation was also coded to match the participant. For example, if a person only participated in 2017 but not 2018, the graph explanation would represent that.

Finally, for individual pages, extraneous headers were removed, and chemical descriptions were revised to ensure they were at an eighth grade, or lower, reading level, with a reading ease of 60% or greater (Fig. 2C). An explanation for why specific chemicals were highlighted was added, and resources for each chemical category were included at the bottom of the page. The community report was three pages, and the individual report was 12 pages (graph explanation page, one page per chemical category (*n* = 9), and 2–3 pages for the table of all individual detections).

## Discussion

In the aftermath of Hurricane Harvey, and widespread flooding, there was substantial concern regarding the types and quantities of chemicals people living in the area might be exposed to [37]. The collection of personal exposure data can help address these concerns, provided it is made available in an accessible format. Therefore, this study assessed community input in the RBRR process, and used the feedback to revise and disseminate community and individual reports. This study assessed community input in prototype reports with the intent of utilizing the feedback for improvement within the report-back process. The added combination of qualitative and quantitative participant feedback from three focus groups allowed for cross-validation of findings to specifically pinpoint what participants found accessible and meaningful, and identified generalizable lessons for developing RBRR.

### Develop a RBRR that is accessible

Overall, as evidenced by scoring of the report as well as verbal feedback, the community report was more well received than the individual report pages. The key differences between the prototype report pages were the community report centered around broad descriptions that reduced the number of technical terms and complex sentences, and contained more graphics, whereas the individual reports were focused on specific chemical categories and therefore contained more terminology that participants may have been unfamiliar with. Similar with the findings from this study, previous research has shown that barriers to understanding scientific materials, even if they were geared for a public audience, include insufficiently explained terminology, and complex sentence structures [38]. Thus, the revised reports reduced the use of technical terms when possible and the overall reading level, and used graphics to increase comprehension [39]. The use of researcher interpretations of the results, the take-home messages, was well received and aided in comprehension. While this study used the Flesch-Kinkaid grade level and Flesch Reading Ease, the SMOG formula (40) has been recommended as a more reliable measure [35].

Graph literacy is another known barrier [41]. While participants preferred the strip chart visualization over a bar chart for individual graphs, accessibility barriers remained. The use of a graphic organizer, a common educational tool [42], was used to improve graph comprehension [7]. Here, the graphic organizer was developed using responsive code, and therefore adapted to each participant. Similarly, the community report used bar charts, which are often preferred, and well understood [43]. Lastly, the challenge remained of reporting the sheer number of chemicals assessed (1,530), with limited regulatory or health values to connect exposure and what that means for human health. For full transparency of results, participants received their full dataset, in table format, at the end of the report. While tables are less preferred over graphical formats, they are well understood [43] and providing graphs for every compound would have exceeded the target RBRR length and would not help inform the participants of key compounds of interest. Therefore, each individual report focused on two chemicals per chemical category as a way to provide information without overwhelming participants with data. The chemicals were selected based on the following criteria, as influenced by focus group feedback: 1) chemicals most frequently detected in the study population; 2) chemicals with known health effects; 3) chemicals of interest to the community and; 4) chemicals associated with known contamination in the area.

Of note, returning results in an online format might make it easier for researchers to add contextual information [18, 44], provide graphical representations for more compounds of interest, and ensure participants are aware of what information the report-back can provide before reading the report.

### Identify the known versus the unknown

It was unfortunate, yet unsurprising that participants had increased difficulty understanding the relevance of the reports to their health. In cases where clinical significance of compounds of interest are unknown, researchers have previously opted to not report individual data, and only report community level data [6]. Researchers have also reported individual level findings while providing inter- and intra- study context [16] when health guidelines are unknown, the approach taken here. These approaches, which provide additional context, remain limited. Exposure assessments for emerging contaminants often lack dose–response relationships, and thus lack regulatory or health standards for the general populace. However, it is necessary to inform participants of their results in a way that allows them to make individual risk calculations and reduce their chemical exposure if they can and if they choose to [12, 45]. Individual chemical descriptions were written to provide information that was known, but also addressed information gaps, or information that is currently unknown, following an adaptation of the Johari Window model [43] to the Known-Unknown classification of risk. Participants, while frustrated that the researchers did not have the answers they wanted, understood that those answers were not available, and were, in fact, unknown. Thus, reports that acknowledge the known from the unknown, and even the unknowable, may resonate more with communities. Here, as in other studies, participants expressed interest in their results, even when the connection to their health was unknown [16, 23, 25].

### Recognize and respect community knowledge and history

Prior work has identified the importance of integrating community voice into RBRR and ensuring RBRR is aligned with the culture and experience of the community [7, 8, 15, 16, 19, 21]. Integrating community knowledge and history goes beyond how the data is translated for RBRR. Notably, participants ranked the report highly for informing behavior change to reduce exposures. However, some exposure reduction strategies may not be possible for some communities due to access, economic, social, or cultural barriers, in which case alternatives need to be proposed [19]. Identifying potential barriers, and providing alternatives, is necessary for useful RBRR. Furthermore, as demonstrated here, different communities have different interests in the type of information being presented, and how it is visualized. Tailoring visualizations and messages for communities is posited to increase participant increase in the report, and may have a greater impact on increasing EHL if the report caters to those interests. The contents of the report may also differ from participants’ lived experiences creating cognitive dissonance and researchers may need to provide additional details about the study methodology. For example, some focus participants expressed that they knew there were more chemicals in the area then was being reported, in which instance the focus group moderator expressed that only organic compounds were looked for, but metals and inorganic metabolites could also be present.

### Set participant expectations

The focus groups were conducted with Houston 3H participants. At the time the focus group occurred many participants had lost and/or forgotten information about the silicone wristbands that was provided when sampling occurred. This likely enabled false expectations for what the study results could and could not tell participants. The reports therefore needed to describe what the study was, and what the resultant data could tell them about their exposures. Contextualizing exposure assessment data in terms of what it meant for human health was a key concern for focus group participants. This was challenging for three reasons: the breadth of chemicals, the current inability to translate wristband results to standard reference values for air or dermal exposure, and a substantial lack of regulatory or health guidelines. To address the lack of exposure assessment information for humans, animal data was occasionally used, yet this was found to be confusing. As discussed above, stating the unknown, e.g., “risks to humans are not known at this time” may be more transparent to the participant. Another approach is to compare exposure measurements to those taken in other studies and in other locations. For instance, how did exposure levels compare in Houston, TX versus rural Oregon? Multiple exposure assessment timepoints can also be utilized to determine if a specific event and/or activity was associated with increased chemical exposure. Lastly, if the sample population is large enough, subgroups can be utilized to provide context to the study. Research indicates that if chemical exposure for one individual or subgroup is much higher than others, they will be more likely to engage in exposure reduction strategies [11].

### Provide resources that report recipients can use to learn more

For usability, the final RBRR contained general information about the sources of some chemicals, ways to reduce chemical exposure, and potential health effects. However, some focus group participants expressed wanting to learn more about the sources and health effects of specific chemicals found, and even regulatory practices. Providing resources is a way to incorporate more information than what is possible to convey in a brief report and gives recipients an opportunity to engage further with the report-back and independently work to increase their EHL. Providing resources can be particularly valuable when conveying the human health effects of specific chemicals. As demonstrated, some participants found the use of animal model or in vitro studies confusing, but if this information is excluded entirely and recipients learn about previous studies autonomously it can lead to increased distrust of the researchers. If this information is provided as a resource, then the participant can choose whether they want to learn about it and determine if they want to incorporate that information in assessing their own risk.

### Limitations

The results of this study are limited by sample size. While three focus groups were conducted, the demographics of focus group participants were not fully representative of the Houston-3H study population, nor the general U.S. population. Furthermore, the concerns from the focus group population may have been different from other communities. This study was conducted in the aftermath of a large-scale environmental disaster and in an area with a high density of industrial activity. The focus groups may also have been susceptible to participation bias in which only Houston-3H participants interested in or concerned about their chemical exposure chose to participate in the focus groups. Additionally, mock data were utilized for the focus groups; responses and engagement with the report may have varied if participants were looking at their own data. Lastly, researchers initially wanted to include results from a post-assessment survey sent with the final report-back, however, due to insufficient participant responses, researchers were unable to test the final version of the report.

## Conclusion

To the coauthor’s knowledge, this was the first time that an exposure assessment study has reported such a large data set back to study participants using silicone wristbands as an exposure assessment tool. Reporting silicone wristband exposure assessment results have the added complication that wristband concentrations are currently not reflective of values that can be compared to reference values or reference doses, and the clinical significance or health relevance of many of the chemicals assessed are not yet known. A total of 1,530 chemicals across nine chemical categories were evaluated at two time points. Given the amount of data presented, and the uncertainty for what those data meant for participants’ health, many participants expressed that the report was interesting, but lacked meaning. Overall, however, participants felt that the reports would be useful, and should be returned to study participants, even in the absence of clinical significance. Future report-backs should include separate documents regarding the sampling tool that was utilized, and how to read graphical representations of the data so that when participants view their report, they are aware of the inherent limitations of exposure assessment and can easily interpret their results. Additionally, addressing community concerns may increase participant interest in the report-backs and make the data more meaningful.

### Supplementary Information

**Additional file 1: Figure S1.** Options and focus group preferences for graphical representation of data. (A) Participants were given four different options for how data could be displayed on the community report: (option 1) average chemical exposure for each chemical category; (option 2) average chemical exposure for each chemical category grouped by community; (option 3) average chemical exposure for each neighborhood grouped by chemical category; and (option 4) average chemical exposure for each chemical category group by exposure to hurricane related flooding. Participants were asked to rank each graph presentation with one as their favorite and four as their least favorite. (B) Aggregate average ranking for graph presentation plus/minus standard deviation. (C) Average ranking for each graph presentation option across each focus group held. **Figure S2**. Percent of participants that preferred error bars across education levels. **Figure S3.** Average scores given for the reports plus/minus standard deviation. Reports were scored on a scale of one to ten, where one is perfect and ten is terrible. **Table S1.** Percentage of times a section/subsection of the community report was circled black or red. Data is presented for the aggregate from all focus groups, and each of the three focus groups held. For Simplicity, section and subsections that were identified by participants with a black or red pen in less than 10% of the study population were removed from the table. Sections and Subsections can be reviewed in Figure 1 A&B. **Table S2.** Percentage of times a section/subsection of the individual report pages were circled black or red. The individual report pages included an Endocrine Disruptors (ED) and Flame Retardants (FR) page. Data is presented for the aggregate from all focus groups, and each of the three focus groups held. For Simplicity, section and subsections that were identified by participants with a black or red pen in less than 10% of the study population were removed from the table. Sections and Subsections can be reviewed in Figure 1 C&D. 

## Data Availability

The datasets generated and analyzed in this current study are not publicly available to protect participant privacy, but are available from the corresponding author on reasonable request.

## Abbreviations

Houston-3H: Houston Hurricane Harvey Health
EHL: Environmental health literacy
RBRR: Report-back of research results
