# Supplementary material for: Integrating participant feedback and concerns to improve community and individual level chemical exposure assessment reports
Source: BMC Public Health. 2023 Sep 6;23:1732. doi: 10.1186/s12889-023-16661-0 (PMC10481616; doi:10.1186/s12889-023-16661-0)
Supplement: Supplementary file 1 — Additional file 1: Figure S1. Options and focus group preferences for graphical representation of data. (A) Participants were given four different options for how data could be displayed on the community report: (option 1) average chemical exposure for each chemical category; (option 2) average chemical exposure for each chemical category grouped by community; (option 3) average chemical exposure for each neighborhood grouped by chemical category; and (option 4) average chemical exposure for each chemical category group by exposure to hurricane related flooding. Participants were asked to rank each graph presentation with one as their favorite and four as their least favorite. (B) Aggregate average ranking for graph presentation plus/minus standard deviation. (C) Average ranking for each graph presentation option across each focus group held. Figure S2. Percent of participants that preferred error bars across education levels. Figure S3. Average scores given for the reports plus/minus standard deviation. Reports were scored on a scale of one to ten, where one is perfect and ten is terrible. Table S1. Percentage of times a section/subsection of the community report was circled black or red. Data is presented for the aggregate from all focus groups, and each of the three focus groups held. For Simplicity, section and subsections that were identified by participants with a black or red pen in less than 10% of the study population were removed from the table. Sections and Subsections can be reviewed in Figure 1 A&B. Table S2. Percentage of times a section/subsection of the individual report pages were circled black or red. The individual report pages included an Endocrine Disruptors (ED) and Flame Retardants (FR) page. Data is presented for the aggregate from all focus groups, and each of the three focus groups held. For Simplicity, section and subsections that were identified by participants with a black or red pen in less than 10% of the study population were removed [file 12889_2023_16661_MOESM1_ESM.docx]

# Supplemental Information

Integrating Participant Feedback and Concerns to Improve Community and Individual Level Chemical Exposure Assessment Reports

Samantha M. Samon ^1^, Michael Barton ^2^, Kim Anderson ^1^, Abiodun Oluyomi ^3, 4^, Melissa Bondy ^5^, Georgina Armstrong ^5^, Diana Rohlman ^6^

^1^Department of Environmental & Molecular Toxicology, Oregon State University, Corvallis, OR

^2^Pacific Northwest Center for Translational Environmental Health Research, Oregon State University, Corvallis, OR

^3^Section of Epidemiology and Population Sciences, Department of Medicine, Baylor College of Medicine, Houston, TX

^4^Gulf Coast Center for Precision Environmental Health, Baylor College of Medicine, Houston, TX

^5^Department of Epidemiology and Population Health, Stanford School of Medicine, Stanford University, Stanford, CA

^6^College of Public Health and Human Sciences, Oregon State University, Corvallis, OR

***Corresponding Author:**

Diana Rohlman

Oregon State University

College of Public Health and Human Services

Weniger Hall 223

103 SW Memorial Place

Corvallis, Oregon 97331, USA

Telephone: (541) 737-4374

Email: [diana.rohlman@oregonstate.edu](mailto:diana.rohlman@oregonstate.edu)


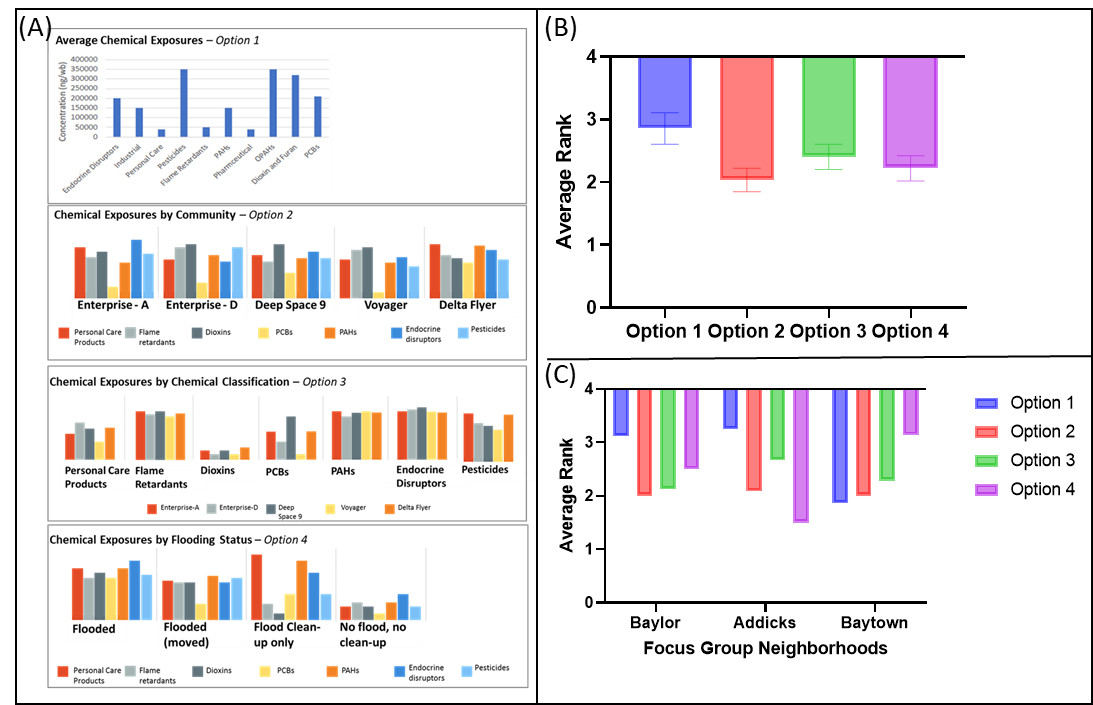


**Figure S1**. *Options and focus group preferences for graphical representation of data*. (A) Participants were given four different options for how data could be displayed on the community report: (option 1) average chemical exposure for each chemical category; (option 2) average chemical exposure for each chemical category grouped by community; (option 3) average chemical exposure for each neighborhood grouped by chemical category; and (option 4) average chemical exposure for each chemical category group by exposure to hurricane related flooding. Participants were asked to rank each graph presentation with one as their favorite and four as their least favorite. (B) Aggregate average ranking for graph presentation plus/minus standard deviation. (C) Average ranking for each graph presentation option across each focus group held.


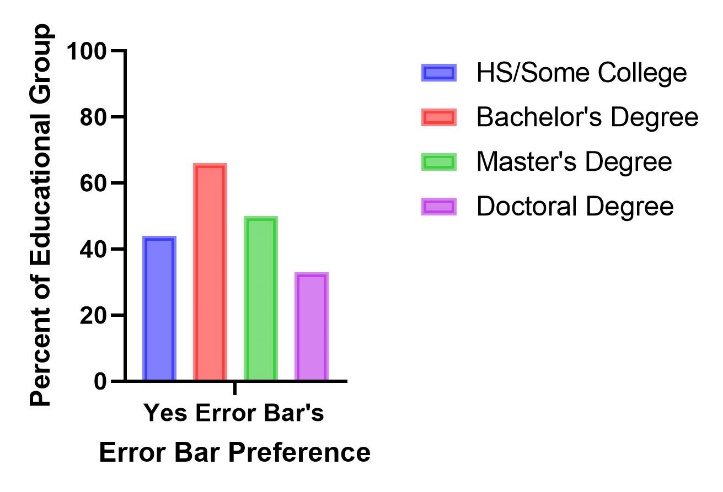


**Figure S2**. Percent of participants that preferred error bars across education levels.

**Figure S3**. *Average scores given for the reports plus/minus standard deviation*. Reports were scored on a scale of one to ten, where one is perfect and ten is terrible.

**Table S1**. *Percentage of times a section/subsection of the community report was circled black or red*. Data is presented for the aggregate from all focus groups, and each of the three focus groups held. For Simplicity, section and subsections that were identified by participants with a black or red pen in less than 10% of the study population were removed from the table. Sections and Subsections can be reviewed in Figure 1 A&B.

| Report Section | Overall | | Baylor | | Addick’s | | Baytown | |
| --- | --- | --- | --- | --- | --- | --- | --- | --- |
|  | Black | Red | Black | Red | Black | Red | Black | Red |
| Pg1_6 | 29.0 | 0 | 11.1 | 0 | 15.4 | 0 | 66.7 | 0 |
| Pg1_6a | 41.9 | 0 | 33.3 | 0 | 15.4 | 0 | 88.9 | 0 |
| Pg1_6b | 29.0 | 0 | 11.1 | 0 | 15.4 | 0 | 66.7 | 0 |
| Pg1_6c | 29.0 | 0 | 11.1 | 0 | 15.4 | 0 | 66.7 | 0 |
| Pg1_6d | 41.9 | 3.2 | 44.4 | 0 | 23.1 | 7.7 | 66.7 | 0 |
| Pg1_7 | 19.4 | 6.5 | 0 | 0 | 15.4 | 15.4 | 44.4 | 0 |
| Pg1_7a | 19.4 | 6.5 | 0 | 0 | 15.4 | 15.4 | 44.4 | 0 |
| Pg1_7b | 38.7 | 58.1 | 22.2 | 77.8 | 30.8 | 53.8 | 66.7 | 44.4 |
| Pg1_7c | 29.0 | 29.0 | 11.1 | 55.6 | 30.8 | 30.8 | 44.4 | 0 |
| Pg1_7d | 54.8 | 19.4 | 33.3 | 22.2 | 53.8 | 30.8 | 77.8 | 0 |
| Pg1_7e | 48.4 | 38.7 | 22.2 | 55.6 | 53.8 | 38.5 | 66.7 | 22.2 |
| Pg1_8 | 16.1 | 0 | 0 | 0 | 15.4 | 0 | 33.3 | 0 |
| Pg1_8a | 22.6 | 6.5 | 22.2 | 11.1 | 7.7 | 0 | 44.4 | 11.1 |
| Pg1_8b | 22.6 | 3.2 | 22.2 | 0 | 7.7 | 0 | 44.4 | 11.1 |
| Pg1_8c | 32.3 | 3.2 | 44.4 | 0 | 15.4 | 0 | 44.4 | 11.1 |
| Pg1_9 | 32.3 | 22.6 | 22.2 | 55.6 | 30.8 | 15.4 | 44.4 | 0 |
| Pg1_10 | 25.8 | 3.2 | 22.2 | 11.1 | 23.1 | 0 | 33.3 | 0 |
| Pg1_11 | 22.6 | 12.9 | 22.2 | 0 | 7.7 | 7.7 | 44.4 | 33.3 |
| Pg1_12 | 12.9 | 12.9 | 33.3 | 11.1 | 0 | 7.7 | 11.1 | 22.2 |
| Pg2_4 | 19.4 | 0 | 33.3 | 0 | 0 | 0 | 33.3 | 0 |
| Pg2_4a | 19.4 | 3.2 | 33.3 | 0 | 0 | 0 | 33.3 | 11.1 |
| Pg2_4b | 25.8 | 0 | 33.3 | 0 | 7.7 | 0 | 44.4 | 0 |
| Pg2_5 | 16.1 | 35.5 | 11.1 | 66.7 | 15.4 | 38.5 | 22.2 | 0 |
| Pg2_6 | 19.4 | 22.6 | 22.2 | 22.2 | 15.4 | 15.4 | 22.2 | 33.3 |
| Pg2_6a | 16.1 | 19.4 | 22.2 | 22.2 | 15.4 | 15.4 | 11.1 | 22.2 |
| Pg2_6b | 19.4 | 29.0 | 22.2 | 22.2 | 15.4 | 15.4 | 22.2 | 55.6 |
| Pg2_6c | 19.4 | 25.8 | 22.2 | 22.2 | 15.4 | 15.4 | 22.2 | 44.4 |
| Pg2_6d | 12.9 | 25.8 | 22.2 | 33.3 | 7.7 | 15.4 | 11.1 | 33.3 |
| Pg2_6e | 22.6 | 51.6 | 22.2 | 22.2 | 30.8 | 92.3 | 11.1 | 22.2 |
| Pg2_7 | 19.4 | 9.7 | 11.1 | 0 | 15.4 | 0 | 33.3 | 33.3 |
| Pg2_8 | 9.7 | 12.9 | 0 | 22.2 | 7.7 | 15.4 | 22.2 | 0 |
| Pg2_9 | 12.9 | 22.6 | 11.1 | 33.3 | 7.7 | 30.8 | 22.2 | 0 |
| Pg2_10 | 16.1 | 3.2 | 11.1 | 11.1 | 7.7 | 0 | 33.3 | 0 |
| Pg2_11 | 16.1 | 0 | 11.1 | 0 | 7.7 | 0 | 33.3 | 0 |
| Pg2_11a | 16.1 | 0 | 11.1 | 0 | 7.7 | 0 | 33.3 | 0 |
| Pg2_11b | 19.4 | 0 | 11.1 | 0 | 15.4 | 0 | 33.3 | 0 |
| Pg2_12 | 45.2 | 0 | 22.2 | 0 | 46.2 | 0 | 66.7 | 0 |
| Pg2_12a | 45.2 | 3.2 | 22.2 | 0 | 46.2 | 0 | 66.7 | 11.1 |
| Pg2_12b | 41.9 | 3.2 | 22.2 | 0 | 38.5 | 0 | 66.7 | 11.1 |
| Pg2_12c | 45.2 | 3.2 | 22.2 | 0 | 46.2 | 7.7 | 66.7 | 0 |
| Pg2_12d | 51.6 | 0 | 33.3 | 0 | 53.8 | 0 | 66.7 | 0 |
| Pg2_13 | 12.9 | 0 | 22.2 | 0 | 0 | 0 | 22.2 | 0 |

**Table S2**. *Percentage of times a section/subsection of the individual report pages were circled black or red*. The individual report pages included an Endocrine Disruptors (ED) and Flame Retardants (FR) page. Data is presented for the aggregate from all focus groups, and each of the three focus groups held. For Simplicity, section and subsections that were identified by participants with a black or red pen in less than 10% of the study population were removed from the table. Sections and Subsections can be reviewed in Figure 1 C&D.

| Report Section | Overall | | Baylor | | | Addick's | | | Baytown | | |  |
| --- | --- | --- | --- | --- | --- | --- | --- | --- | --- | --- | --- | --- |
|  | Black | Red | | Black | Red | | Black | Red | | Black | Red | |
| ED_1 | 12.9 | 0 | | 0 | 0 | | 23.1 | 0 | | 11.1 | 0 | |
| ED_2 | 12.9 | 0 | | 0 | 0 | | 15.4 | 0 | | 22.2 | 0 | |
| ED_3 | 45.2 | 0 | | 44.4 | 0 | | 61.5 | 0 | | 22.2 | 0 | |
| ED_4 | 16.1 | 0 | | 0 | 0 | | 30.8 | 0 | | 11.1 | 0 | |
| ED_5a | 41.9 | 6.5 | | 22.2 | 22.2 | | 53.8 | 0 | | 44.4 | 0 | |
| ED_5b | 25.8 | 6.5 | | 0 | 11.1 | | 38.5 | 7.7 | | 33.3 | 0 | |
| ED_6 | 38.7 | 32.3 | | 11.1 | 33.3 | | 46.2 | 53.8 | | 55.6 | 0 | |
| ED_7a | 29.0 | 32.3 | | 33.3 | 22.2 | | 38.5 | 46.2 | | 11.1 | 22.2 | |
| ED_7b | 19.4 | 19.4 | | 22.2 | 11.1 | | 15.4 | 30.8 | | 22.2 | 11.1 | |
| ED_7c | 22.6 | 19.4 | | 33.3 | 11.1 | | 15.4 | 30.8 | | 22.2 | 11.1 | |
| ED_8a | 41.9 | 6.5 | | 33.3 | 0 | | 53.8 | 15.4 | | 33.3 | 0 | |
| ED_8b | 41.9 | 6.5 | | 33.3 | 0 | | 53.8 | 15.4 | | 33.3 | 0 | |
| ED_9a | 58.1 | 19.4 | | 66.7 | 33.3 | | 69.2 | 23.1 | | 33.3 | 0 | |
| ED_9b | 58.1 | 19.4 | | 66.7 | 33.3 | | 69.2 | 23.1 | | 33.3 | 0 | |
| ED_9c | 61.3 | 9.7 | | 66.7 | 11.1 | | 76.9 | 15.4 | | 33.3 | 0 | |
| ED_9d | 58.1 | 9.7 | | 66.7 | 11.1 | | 69.2 | 15.4 | | 33.3 | 0 | |
| ED_9e | 58.1 | 12.9 | | 66.7 | 22.2 | | 69.2 | 15.4 | | 33.3 | 0 | |
| ED_9f | 61.3 | 12.9 | | 66.7 | 22.2 | | 76.9 | 15.4 | | 33.3 | 0 | |
| ED_10 | 29.0 | 6.5 | | 33.3 | 11.1 | | 38.5 | 0 | | 11.1 | 11.1 | |
| FR_3 | 35.5 | 0 | | 22.2 | 0 | | 53.8 | 0 | | 22.2 | 0 | |
| FR_4 | 25.8 | 0 | | 22.2 | 0 | | 30.8 | 0 | | 22.2 | 0 | |
| FR_4a | 35.5 | 0 | | 22.2 | 0 | | 46.2 | 0 | | 33.3 | 0 | |
| FR_4b | 35.5 | 3.2 | | 33.3 | 11.1 | | 38.5 | 0 | | 33.3 | 0 | |
| FR_4c | 29.0 | 6.5 | | 22.2 | 0 | | 38.5 | 15.4 | | 22.2 | 0 | |
| FR_4d | 29.0 | 6.5 | | 33.3 | 11.1 | | 30.8 | 7.7 | | 22.2 | 0 | |
| FR_4e | 32.3 | 6.5 | | 33.3 | 11.1 | | 38.5 | 7.7 | | 22.2 | 0 | |
| FR_4f | 25.8 | 3.2 | | 22.2 | 11.1 | | 30.8 | 0 | | 22.2 | 0 | |
| FR_5 | 25.8 | 35.5 | | 11.1 | 33.3 | | 30.8 | 53.8 | | 33.3 | 11.1 | |
| FR_6 | 22.6 | 35.5 | | 11.1 | 33.3 | | 23.1 | 53.8 | | 33.3 | 11.1 | |
| FR_7 | 22.6 | 35.5 | | 11.1 | 33.3 | | 23.1 | 53.8 | | 33.3 | 11.1 | |
| FR_8 | 48.4 | 6.5 | | 66.7 | 0 | | 53.8 | 15.4 | | 22.2 | 0 | |
| FR_8a | 48.4 | 6.5 | | 66.7 | 0 | | 53.8 | 15.4 | | 22.2 | 0 | |
| FR_8b | 51.6 | 6.5 | | 66.7 | 0 | | 53.8 | 15.4 | | 33.3 | 0 | |
| FR_8c | 51.6 | 6.5 | | 66.7 | 0 | | 53.8 | 15.4 | | 33.3 | 0 | |
| FR_8d | 54.8 | 6.5 | | 66.7 | 0 | | 53.8 | 15.4 | | 44.4 | 0 | |
| FR_8e | 51.6 | 6.5 | | 66.7 | 0 | | 53.8 | 15.4 | | 33.3 | 0 | |
| FR_8f | 54.8 | 6.5 | | 66.7 | 0 | | 53.8 | 15.4 | | 44.4 | 0 | |
| FR_9 | 16.1 | 3.2 | | 0 | 0 | | 30.8 | 0 | | 11.1 | 11.1 | |
